# Longitudinal Descriptive Analysis of Dynamic Changes in Safety Concerns in Japanese Risk Management Plans for Medicinal Products Over 8 Years After Approval

**DOI:** 10.1007/s43441-025-00801-2

**Published:** 2025-07-30

**Authors:** Chieko Ishiguro, Mahiro Sazawa, Takahiro Nonaka

**Affiliations:** 1 Laboratory of Clinical Epidemiology, Department of Data Science, Center for Clinical Sciences, Japan Institute for Health Security, 1-21-1 Toyama, Shinjuku-ku, Tokyo, 162-8655 Japan; 2 Department of Clinical Research Governance, Graduate School of Medicine, The University of Tokyo Hospital, The University of Tokyo, Tokyo, Japan

**Keywords:** Risk management plan, Pharmacovigilance, Drug safety, Regulatory science, Japan

## Abstract

**Introduction:**

A Japanese risk management plan (RMP) is a proactive planning tool for managing safety concerns (important identified risk [IIR], important potential risk [IPR], and important missing information [IMI]) for each drug and is continuously updated. However, no studies have examined the dynamic changes of safety concerns in RMPs throughout the drug lifecycle.

**Methods:**

We conducted a longitudinal descriptive analysis of safety concerns in RMPs of drugs approved for new active ingredients in 2014 in Japan. We compared safety concerns in RMPs between the first version at approval and the latest version 8 years after the approval date using the Sankey diagram. We also investigated the evidence for RMP changes.

**Results:**

This analysis included 38 drugs, whose first version RMPs included 155 IIRs, 119 IPRs, and 59 IMIs. Among them, all IIRs and 88% of the IPRs and the IMIs remained in the latest version of the RMPs 8 years after the approval date. During follow-up, 29 IIRs, 20 IPRs, and 3 IMIs were newly added, 14 IPRs were upgraded to IIRs, and 7 IMIs were deleted; thus, the final numbers of IIRs, IPRs, IMIs were 198, 125, and 55, respectively. Evidence for RMP changes was more often obtained from pharmacovigilance activities than from clinical/non-clinical studies conducted for additional approvals.

**Conclusions:**

Most of the safety concerns identified at the first approval remained over 8 years, and the number of IIRs and IPRs tended to increase after approval. Most of the RMP changes were based on pharmacovigilance activities.

**Supplementary Information:**

The online version contains supplementary material available at 10.1007/s43441-025-00801-2.

## Introduction

In Japan, a risk management plan (RMP) has been required for all drugs submitted for marketing authorization since April 2013 [1, 2]. An RMP includes pharmacovigilance and risk minimization plans addressing each safety concern regarding an individual drug. It clearly summarizes consistent risk management strategies from drug development to post-marketing and ensures that evaluations are carried out regularly and reliably based on the progress of post-marketing studies and risk minimizations. The aim is to enhance and strengthen post-marketing safety measures by publishing RMPs and sharing their content with healthcare professionals [3]. The first version of the RMP per drug is created based on data from clinical and non-clinical studies before approval; thereafter, it is continuously updated with the latest scientific knowledge, including post-marketing data. The RMP is deleted when the approval conditions for RMP implementation are deemed to have been fulfilled by a re-examination review [4], which is mandatory for new drugs and usually occurs eight (non-orphan drugs) or 10 years (orphan drugs) after approval in case of a new active ingredient [5]. When there is an additional approval during the re-examination period, the re-examination period re-starts from that point and the RMP will be extended accordingly.

Safety concerns described in RMPs are categorized into three types: important identified risks (IIRs), important potential risks (IPRs), and important missing information (IMI). IIRs are prominent adverse events associated with the drug based on sufficient evidence, whereas IPRs are prominent adverse events that are suspected to be associated with the drug but lack sufficient support from available clinical data. IMI is critical data needed to estimate the post-marketing safety of a drug that is not sufficient at the time of RMP planning [1].

Pharmacovigilance and risk minimization activities are planned to address each safety concern during the post-marketing phase. If the plans are working well, an IPR is expected to be upgraded an IIR or removed from the RMP, and IMIs are expected to disappear. Thus, the degree of dynamic change of safety concerns can be an important indicator for inferring whether the RMP is working well. Previous studies on RMPs have focused on their initial versions [6, 7], showing the distribution of IIRs and IPRs for RMPs published at a single point in time [8], or showing the transition of safety concerns for each product [9]. However, no studies have systematically and longitudinally examined the dynamic changes in safety concerns in RMPs during a prolonged post-marketing period. Therefore, this study aimed to clarify the dynamic changes of safety concerns in Japanese RMPs after approval using a systematic and longitudinal approach.

## Materials and Methods

### Data Sources

Three kinds of data sources were used in this study. First, information on all new drugs approved in Japan was identified using the list of new drug approvals on the official website of the Pharmaceuticals and Medical Devices Agency (PMDA) [10]. The list provided an outline of approval information such as drug name, approval date, and approval category. Second, additional details were obtained from the new drug review report published on the PMDA website [11]. Third, a dataset containing information on all RMPs published in Japan from January 2014 to December 2022 (including previous versions of RMPs) was provided by the EPS corporation (Tokyo, Japan), a company that collected all RMPs published on the PMDA website since 2013 [12]. Access to this dataset was necessary because the PMDA website only provides the current ongoing RMP with the latest version.

### Target Cohort and Follow-up Period

The target cohort included drugs (human medicinal products) containing new active ingredients that were approved in fiscal year 2014 (April 2014 to March 2015) identified on the PMDA website [10]. The exclusion criteria were: (1) drugs with a new drug application date before April 1, 2013, because an RMP was not required for new drugs applications submitted before April 1, 2013, (2) drugs without RMPs (approved but not marketed), and (3) drugs whose RMPs were removed within 8 years of the approval date (marketing suspension or approval withdrawn). The start of the follow-up was the approval date of each target drug, and the end date was exactly 8 years after the approval date.

### Safety Concerns in RMPs at Baseline and After 8 Years

Safety concerns (IIRs, IPRs, and IMI) at baseline were identified from the first version of the RMP of each target drug. Safety concerns at the end of follow-up were identified from the latest version of the RMP at the end date of the follow-up period.

### Evidence for RMP Changes

In general, RMPs are revised based on information from routine or additional pharmacovigilance activities, in which case the reasons for the revisions are not disclosed. However, they may also be revised based on information from clinical trials or non-clinical studies conducted for an additional approval application, in which case the reasons for the revisions are described in the PMDA review reports. Therefore, we investigated the PMDA review reports of the additional approvals during follow-up for each target drug to confirm whether there was a description of the evidence for changes to the RMP (addition, deletion/upgrade, or removal) based on information from clinical trials or non-clinical studies conducted for the submission of the additional approval. If there was such a description, we defined the evidence for changes to the RMP as information from clinical trials or non-clinical studies conducted for additional approvals; otherwise, we defined it as information from pharmacovigilance activities.

### Statistical Analysis

We described the baseline characteristics of the target drugs (drug class based on ATC classification and period of re-examination), which information was identified from the first version of the RMPs, and the characteristics of the target drugs during follow-up (number of additional approvals, type of additional approvals). We also described the baseline characteristics of the safety concerns described in the RMPs of the target drugs. A Sankey diagram was used to describe the dynamic changes in safety concerns in RMPs for target drugs during the follow-up period. The average number of safety concerns (IIRs, IPRs, and IMI) per RMP at baseline and at the end of follow-up was calculated. We conducted a subgroup analysis according to the presence of an additional approval during follow-up. We also described the evidence for RMP changes (clinical/non-clinical trial, pharmacovigilance activity). Analyses were performed using R version 4.1.2 (R Foundation for Statistical Computing, Vienna, Austria).

## Results

**Figure 1:**
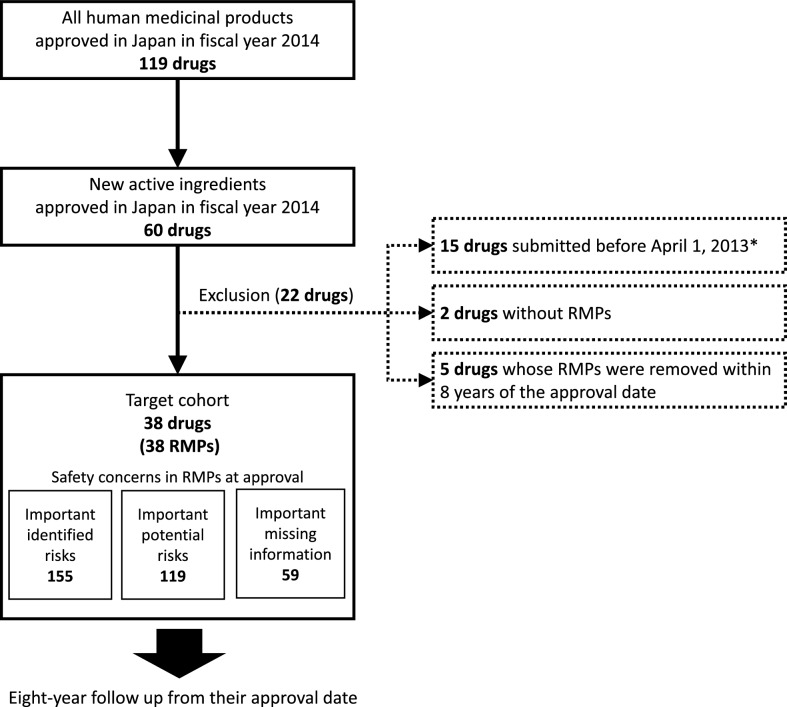
Flow diagram of the study design. *RMP* Risk management plan. *RMPs were not imposed on products submitted prior to April 1, 2013

We identified 38 drugs satisfying the inclusion and exclusion criteria and 333 safety concerns in their respective RMPs at baseline, comprising 155 IIRs, 119 IPRs, and 59 IMIs (Fig. 1). The product information of the 38 included drugs is listed in the Supplemental Table 1. The characteristics of the target drugs at baseline and during the follow-up period are shown in Table 1. The characteristics of safety concerns in RMPs of the target drugs at approval are shown in Table 2.

**Table 1:** Characteristics of the target drugs at baseline and during the follow-up period

	Number of drugs (%)
Total	38
Baseline (at their first approval)	
Drug class*	
Alimentary tract and metabolism	9 (24)
Blood and blood forming organs	5 (13)
Cardiovascular system	1 (3)
Dermatological	1 (3)
Anti-infective for systemic use	1 (3)
Anti-neoplastic and immunomodulating agents	15 (39)
Nervous system	1 (3)
Respiratory system	1 (3)
Sensory organs	1 (3)
Various	3 (8)
Type of drug	
Non-orphan drug	22 (58)
Orphan drug	16 (42)
During 8-year follow-up period	
Number of additional approvals	
None	23 (61)
One	13 (34)
Two	0 (0)
Three or more	2 (5)
Type of additional approvals	
New indication only	4 (11)
New indication and new dosage	9 (24)
New indication and new dosage form	2 (5)

*Classified by Anatomical Therapeutic Chemical codes

**Table 2:** Characteristics of safety concerns in risk management plans at approval

	Number of safety concerns
Total	333
Important identified risk*	155
Certain infectious and parasitic diseases (A00–B99)	13
Neoplasms, diseases of the blood and blood-forming organs and certain disorders involving the immune mechanism (C00–D89)	19
Endocrine, nutritional and metabolic diseases (E00–E90)	13
Mental and behavioral disorders (F00–F99)	1
Nervous system diseases (G00–G99)	3
Eye, adnexa, ear, and mastoid process diseases (H00–H95)	3
Circulatory system diseases (I00–I99)	17
Respiratory system diseases (J00–J99)	13
Digestive system diseases (K00–K93)	17
Skin and subcutaneous tissue diseases (L00–L99)	7
Musculoskeletal system and connective tissue diseases (M00–M99)	3
Genitourinary system diseases (N00–N99)	7
Congenital malformations, deformations, and chromosomal abnormalities (Q00–Q99)	1
Symptoms, signs, and abnormal clinical and laboratory findings, not elsewhere classified (R00–R99)	15
Injury, poisoning, and certain other consequences of external causes (S00–T98)	18
Production of anti-drug antibodies	1
Interaction	2
Other	2
Important potential risk*	119
Certain infectious and parasitic diseases (A00–B99)	7
Neoplasms, diseases of the blood and blood-forming organs and certain disorders involving the immune mechanism (C00-D89)	20
Endocrine, nutritional and metabolic diseases (E00–E90)	11
Mental and behavioral disorders (F00–F99)	4
Nervous system diseases (G00–G99)	4
Eye, adnexa, ear, and mastoid process diseases (H00–H95)	4
Circulatory system diseases (I00–I99)	10
Respiratory system diseases (J00–J99)	4
Digestive system diseases (K00–K93)	6
Skin and subcutaneous tissue diseases (L00–L99)	2
Musculoskeletal system and connective tissue diseases (M00–M99)	4
Genitourinary system diseases (N00–N99)	6
Certain conditions originating in the perinatal period (P00–P96)	2
Congenital malformations, deformations, and chromosomal abnormalities (Q00–Q99)	3
Symptoms, signs and abnormal clinical and laboratory findings, not elsewhere classified (R00–R99)	7
Injury, poisoning and certain other consequences of external causes (S00–T98)	9
Production of anti-drug antibodies	5
Interaction	6
Other	5
Important missing information	59
Patient with hepatic dysfunction	9
Patient with renal dysfunction	7
Patient with other baseline diseases**	12
Concomitant drug	8
Long-term administration	7
Elderly patients	5
Children	4
Japanese	2
Other***	5

*Classified by ICD-10 codes. **Various baseline diseases except for hepatic and renal dysfunction. ***Concerns that had one each (effect of aging, change of route of administration, drug switching, pregnant women or lactating women, high dose use)

**Figure 2:**
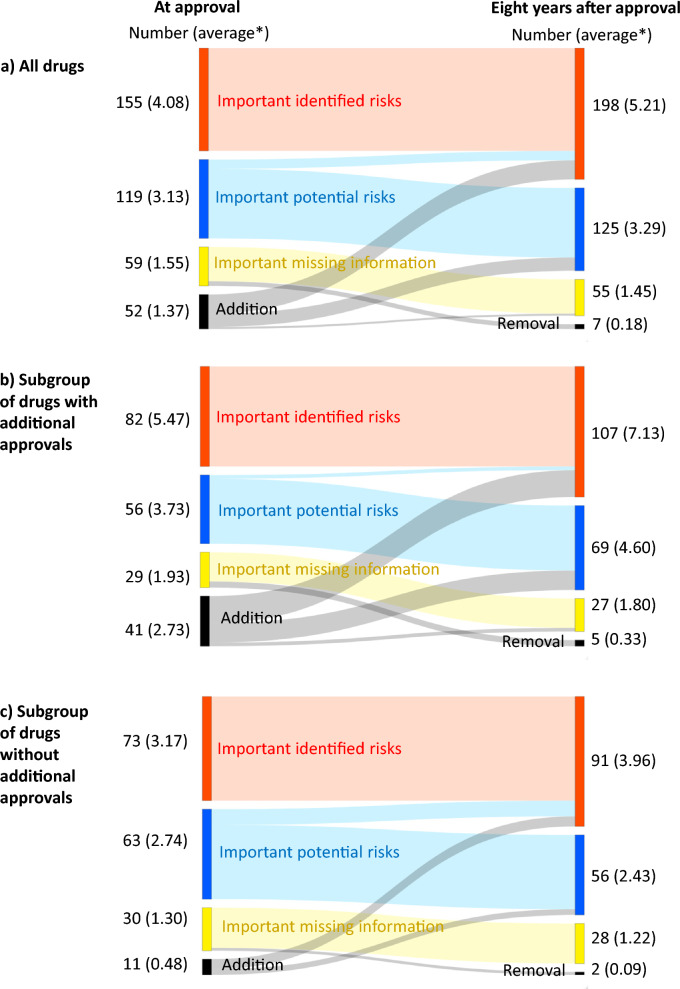
Eight-year journey of safety concerns in risk management plans after initial approval. *Average per risk management plan

The dynamic changes in safety concerns in RMPs for the target drugs during the follow-up period are shown in Fig. 2. The journey of safety concerns of all target drugs (Fig. 2 a, Table 3) showed that all 155 IIRs at baseline remained. During the follow-up period, 29 IIRs were newly added, 14 were added due to upgrades from IPRs to IIRs, none were deleted, and the final number of IIRs was 198. Of the 119 IPRs at baseline, 105 remained (88.2%), 20 were newly added, 14 were deleted owing to an upgrade to IIRs, and the final number of IPRs was 125. Of the 59 IMIs, 52 remained (88.1%), 3 were newly added, 7 were removed, and the final number was 55. Both the average number of IIRs (4.08/RMP at baseline, 5.21/RMP at the end of follow-up) and the average number of IPRs (3.13/RMP at baseline, 3.29/RMP at the end of follow-up) were higher at the end of follow-up than at baseline, while the average IMI number was slightly lower (1.55/RMP at baseline, 1.45/RMP at the end of follow-up). The average number of safety concerns newly added or removed from RMPs during the 8-year follow-up period was 1.37/RMP and 0.18/RMP, respectively. In the subgroup analysis (Fig. 2 b and c), both the average number of safety concerns newly added (2.73/RMP) and removed (0.33/RMP) were higher in the subgroup with additional approvals during the follow-up than those in the other (0.48/RMP for addition, 0.09/RMP for removal). The background characteristics of each subgroup are shown in Supplemental Table 2. The information on what changes (additions, deletions, and removal of safety concerns) to RMPs were based on was more often obtained from pharmacovigilance activities than from clinical/non-clinical trials (Table 3).

**Table 3:** Evidence for changes of safety concerns in risk management plans during 8 years after approval

Safety concerns 8 years after approval	Number	Evidence of RMP changes	
		Clinical or non-clinical trials*	Pharmacovigilance activities
Total	378		
Important identified risk	198		
Remained	155	–	–
Added	29	7	22
Upgraded from important potential risk	14	1	13
(Removed from RMP)	(0)	–	–
Important potential risk	125		
Remained	105	–	–
Added	20	3	17
(Deleted owing to upgrading)	(14)	(1)	(13)
(Removed from RMP)	(0)	–	–
Important missing information	55		
Remained	52	–	–
Added	3	3	0
(Removed from RMP)	(7)	(0)	(7)

*RMP* risk management plan

*Clinical or non-clinical trials coundcted for the submissitons of additional approvals during the follow-up periods. One important potential risk was added based on a non-clinical study (animal experiment), others were added based on clinical trials

## Discussion

Our longitudinal analysis of RMP safety concerns for 38 new drugs approved in 2014 identified 155 IIRs, 119 IPRs, and 59 IMIs in the first edition. After 8 years, most IIRs and over 88% of the IPRs and IMIs remained; 29 IIRs, 20 IPRs, and 3 IMIs were added; 14 IPRs were upgraded to IIRs; and 7 IMIs were removed. More dynamic changes were especially noted for products with additional approvals, but most of the evidence for these changes was based on pharmacovigilance data rather than on clinical trial data.

These results suggested that pharmacovigilance activities occupy a vital role in safety assessment in the post-marketing phase in Japan. First, the number of IIRs added after approval (where no signal for such risks was found in pre-approval clinical trials) accounted for about 15% (29/198) of all IIRs. Second, the changes of newly added IIRs or IPRs and IPRs upgraded to IIRs were based on evidence gathered through pharmacovigilance activities rather than from clinical trials. Third, all new IMIs added during the follow-up period were added due to the results of clinical trials conducted for approvals of additional indications. Lastly, 7 IMIs were removed from RMPs during follow-up based on pharmacovigilance data. All these results not only highlight the importance of pharmacovigilance activities but also the limitations of clinical trial conclusions for use in safety assessments. It is known that there are five limitations to clinical trials from the perspective of safety evaluation: too few (small number of patients), too simple (simple design), too brief (short administration period), too median-aged (no children or elderly patients), and too narrow (narrow range of indications) [13]. Our results show that these limitations of clinical trials were complemented by pharmacovigilance activities.

All IIRs in RMPs at first approval remained for 8 years. There is a definition for the inclusion of IIRs in RMPs, but no definition for the removal of IIRs from RMPs in the Japanese RMP guideline [1]. Meanwhile, the EU RMP guideline, Guideline on good pharmacovigilance practices Module V, allows for the deletion of IIRs under certain circumstances; for example, when the risk is fully characterized and appropriately managed, IIRs may be removed from the safety specification [14]. The content of this guideline was revised in 2017; since then, the number of safety concerns in existing EU RMPs decreased [9]. According to the EU guidelines, IIRs can be removed if the risk is adequately characterized by pharmacovigilance activities and adequately managed by risk minimization activities. It is unclear whether IIRs remained in Japanese RMPs because pharmacovigilance and risk minimization activities have not been functional, or because they have been functional but have not warranted the removal during the re-examination period. In either case, the current situation is problematic; in the former case, sufficient evaluations have not been carried out and risks have not been minimized; in the latter case, RMPs were not updated regularly and did not focus on important aspects of patient safety in clinical practice based on the latest information. Therefore, it is imperative that appropriate pharmacovigilance and risk minimization activities are planned, implemented, and evaluated, and that the need for IIRs is reconsidered based on the latest information.

Of the IPRs present at first approval, 88% remained as IPRs, 12% were changed to IIRs, and none were removed from the RMPs during the 8 year study period. IPRs are those safety concerns for which a causal relationship could not be established at the time of approval owing to a lack of evidence, and for which further information continues to be gathered through routine and additional pharmacovigilance activities. In this study, half of the target drugs were non-orphan drugs and had completed the re-examination period at the end of the follow-up period; i.e., even though all pharmacovigilance activities necessary to address safety concerns had been completed, most IPRs remained. These results could indicate that additional or routine pharmacovigilance activities planned at the time of approval were insufficient to assess causality, or that these activities did not show causality but the IPRs remained because there are no criteria for their removal from the RMPs. The former issue may be due to the fact that post-marketing studies without a control group through primary data collection account for about 80% of all additional pharmacovigilance activities in Japan [15]. Because potential risks identified in clinical trials often require quantitative analysis with a control group to assess causality in the post-marketing setting, causality is challenging to assess based on the results of such studies without a control group. Recently, the number of health information databases available for epidemiological studies in Japan has been increasing [16]. It is necessary to plan additional and more appropriate pharmacovigilance activities using these databases to generate evidence. On the other hand, regarding the latter issue, similar to the IIR issue, a standard procedure for deletion may be needed.

Subgroup analyses showed a trend toward more changes in IIRs, IPRs, and IMIs in the group with additional approvals. However, only a limited number of changes were based on clinical trials conducted for additional approvals. These results may be because drugs with additional approvals have more opportunities to revise their RMPs and because more safety information is available from safety pharmacovigilance activities since the additional indications increase the number of patients using these drugs.

There are several limitations to this study. First, only drugs with new active ingredients approved in 2014 (immediately after the introduction of RMPs in Japan) were included because we needed at least 8 years of follow-up. However, we believe that the results of this study could be extrapolated to drugs approved in other years because the procedure for IIRs, IPRs, and IMIs has currently not changed, and because target drugs with various drug classes were included. Second, the 8-year follow-up period meant that orphan drugs could not be followed up to the end of their re-examination period (10 years). In addition, we were unable to obtain the results of PMDA re-examination reviews for most drugs. Marketing authorization holders generally must submit a re-examination application within 3 months of the end of the re-examination period and the PMDA re-examination review process takes additional time (usually more than one year) from submission. Therefore, further long-term research is needed to comprehensively assess the impact of the re-examination review process on safety concerns in RMPs. Third, the details of the information supporting RMP changes could not be determined because the reasons for RMP changes were not public. Further research and public information on the drivers of RMP changes are needed to improve the functioning of the J-RMP.

## Conclusion

Most of the RMP changes were based on pharmacovigilance activities, suggesting that pharmacovigilance activities play an important role in drug safety assessment in Japan. On the other hand, most safety concerns at the first approval of an active ingredient remained for 8 years, and the number of concerns tended to increase after approval. It might be necessary to plan more appropriate pharmacovigilance and risk minimization activities, evaluate their suitability, and reconsider the need for listing them in the RMP during the re-examination period. Further research is needed on the long-term dynamic changes of safety concerns in RMPs.

## Supplementary Information

Below is the link to the electronic supplementary material.

Supplementary file1 (DOCX 37 KB)

## Data Availability

Data is provided within the manuscript, references, and supplementary information files.
