# Supplementary material for: Longitudinal Descriptive Analysis of Dynamic Changes in Safety Concerns in Japanese Risk Management Plans for Medicinal Products Over 8 Years After Approval
Source: Ther Innov Regul Sci. 2025 Jul 30;59(6):1463–71. doi: 10.1007/s43441-025-00801-2 (PMC12579639; doi:10.1007/s43441-025-00801-2)
Supplement: Supplementary file 1 — Supplementary file1 (DOCX 37 KB) [file 43441_2025_801_MOESM1_ESM.docx]

**Supplementary Table 1. Target drugs**

| Product name | Active ingredient | Ophan drug  (at approval) | Indication  (at approval) | Additional approvals during 8year |
| --- | --- | --- | --- | --- |
| Adempas | Riociguat | ✓ | Chronic thromboembolic pulmonary hypertension | Yes |
| TREPROST | Treprostinil |  | Pulmonary arterial hypertension | No |
| Efient | Prasugrel Hydrochloride |  | Ischemic heart disease | Yes |
| Tysabri | Natalizumab (Genetical Recombination) | ✓ | Multiple Sclerosis | No |
| Tapenta | Tapentadol Hydrochloride |  | Cancer pain | No |
| Lusefi | Luseogliflozin Hydrate |  | Type 2 diabetes | No |
| DEBERZA | Tofogliflozin Hydrate |  | Type 2 diabetes | No |
| Xtandi | Enzalutamide |  | Prostate cancer | Yes |
| Tivicay | Dolutegravir sodium | ✓ | HIV (human immunodeficiency virus) infection | No |
| ANORO | Umeclidinium Bromide / Vilanterol Trifenatate |  | Chronic obstructive pulmonary disease (COPD) | No |
| Rapalimus | Sirolimus | ✓ | Lymphangioleiomyomatosis | Yes |
| CANAGLU | Canagliflozin Hydrate |  | Type 2 diabetes | Yes |
| VPRIV | Velaglucerase Alfa (Genetical Recombination) | ✓ | Gaucher disease | No |
| Nicystagon | Cysteamine Bitartrate | ✓ | Nephrocystinosis | No |
| ZYTIGA | Abiraterone Acetate |  | Prostate cancer | Yes |
| Jevtana | Cabazitaxel Acetonate |  | Prostate cancer | No |
| JAKAVI | Ruxolitinib Phosphate | ✓ | Myelofibrosis | Yes |
| ALECENSA | Alectinib Hydrochloride | ✓ | Non-small cell lung cancer | Yes |
| OPDIVO | Nivolumab (Genetical Recombination) | ✓ | Malignant melanoma | Yes |
| Byclot | Freeze-dried activated human blood coagulation factor VII concentrate containing factor X | ✓ | Patients with inhibitors of blood coagulation factor VIII or IX factor | Yes |
| ALPROLIX | Eftrenonacog Alfa (Genetical Recombination) |  | Patients with deficiency of blood coagulation factor IX | No |
| Fomepizole | Fomepizole |  | Ethylene glycol and methanol poisoning | No |
| GLANATEC | Ripasudil Hydrochloride Hydrate |  | Glaucoma | No |
| G-LASTA | Pegfilagrastim (Genetical Recombination) |  | Febrile neutropenia | Yes |
| AGRYLIN | Anagrelide Hydrochloride Hydrate | ✓ | Essential thrombocythemia | No |
| MabCampath | Alemtuzumab (Genetical Recombination) | ✓ | Chronic lymphocytic leukemia | Yes |
| Bosulif | Bosutinib Hydrate | ✓ | Chronic myeloid leukemia (CML) | Yes |
| ZANOSAR | Streptozocin | ✓ | Pancreatic and gastrointestinal neuroendocrine tumors | No |
| Takecab | Vonoprazan Fumarate |  | Gastric and duodenal ulcer | No |
| METHYLENE BLUE | Methylthioninium Chloride Hydrate |  | Addictive methemoglobinemia | No |
| BEPIO | Benzoyl Peroxide |  | Acne vulgaris | No |
| Therapeutic mite allergen extract subcutaneous injection “Torii” | Dermatophagoides farinae extract/Dermatophagoides pteronyssinus extract |  | Allergic rhinitis and bronchial asthma with tick antigen | No |
| Cosentyx | Secukinumab (Genetical Recombination) |  | Plaque psoriasis and psoriatic arthritis | Yes |
| Orfadin | Nitisinone |  | Tyrosinemia type 1 | No |
| Jardiance | Empagliflozin |  | Type 2 diabetes | Yes |
| Vimizim | Elosulfase Alfa (Genetical Recombination) | ✓ | Mucopolysaccharide IV A type | No |
| ZELBORAF | Vemurafenib | ✓ | Malignant melanoma | No |
| ELOCTATE | Efraloctocog Alfa (Genetical Recombination) |  | Patients with deficiency of blood coagulation factor VIII | No |

**Supplementary Table 2. Characteristics of the target drugs by subgroup at baseline and during the follow-up period**

|  | Number of drugs (%) | |
| --- | --- | --- |
|  | Drugs  with additional approvals | Drugs without additional approvals |
| Total | 15 | 23 |
| Baseline (at approval) |  |  |
| Drug class* |  |  |
| Alimentary Tract And Metabolism | 2 (13) | 7 (30) |
| Blood And Blood Forming Organs | 2 (13) | 3 (13) |
| Cardiovascular System | 1 (7) | 0 (0) |
| Dermatologicals | 0 (0) | 1 (4) |
| Anti-infectives For Systemic Use | 0 (0) | 1 (4) |
| Antineoplastic And Immunomodulating Agents | 10 (67) | 5 (22) |
| Nervous System | 0 (0) | 1 (4) |
| Respiratory System | 0 (0) | 1 (4) |
| Sensory Organs | 0 (0) | 1 (4) |
| Various | 0 (0) | 3 (13) |
| Period of re-examination |  |  |
| 8 years (Except for orphan drugs) | 7 (47) | 15 (65) |
| 10 years (Orphan drugs) | 8 (53) | 8 (35) |
|  |  |  |
| During 8-year follow-up period |  |  |
| Number of additional approvals |  |  |
| None | 0 (0) | 23 (100) |
| 1 | 13 (87) | － |
| 2 | 0 (0) | － |
| 3 or more | 2 (13) | － |
| Type of additional approvals |  |  |
| New indication only | 4 (27) | － |
| New indication and new dosage | 9 (60) | － |
| New indication and new dosage form | 2 (13) | － |

*Classified by Anatomical Therapeutic Chemical codes
